# A Procalcitonin-Based Algorithm to Guide Antibiotic Therapy in Secondary Peritonitis following Emergency Surgery: A Prospective Study with Propensity Score Matching Analysis

**DOI:** 10.1371/journal.pone.0090539

**Published:** 2014-03-04

**Authors:** Ting-Shuo Huang, Shie-Shian Huang, Yu-Chiau Shyu, Chun-Hui Lee, Shyh-Chuan Jwo, Pei-Jer Chen, Huang-Yang Chen

**Affiliations:** 1 The Graduate Institute of Clinical Medicine, National Taiwan University College of Medicine, Taipei, Taiwan; 2 Division of General Surgery, Department of Surgery, Chang Gung Memorial Hospital, Keelung Branch, Keelung, Taiwan; 3 Division of Infectious Diseases, Department of Medicine, Chang Gung Memorial Hospital, Keelung Branch, Keelung, Taiwan; 4 Institute of Biopharmaceutical Sciences, National Yang-Ming University, Taipei, Taiwan; 5 Department of Education and Research, Taipei City Hospital, Taipei, Taiwan; University of Florida College of Medicine, United States of America

## Abstract

**Background:**

Procalcitonin (PCT)-based algorithms have been used to guide antibiotic therapy in several clinical settings. However, evidence supporting PCT-based algorithms for secondary peritonitis after emergency surgery is scanty. In this study, we aimed to investigate whether a PCT-based algorithm could safely reduce antibiotic exposure in this population.

**Methods/Principal Findings:**

From April 2012 to March 2013, patients that had secondary peritonitis diagnosed at the emergency department and underwent emergency surgery were screened for eligibility. PCT levels were obtained pre-operatively, on post-operative days 1, 3, 5, and 7, and on subsequent days if needed. Antibiotics were discontinued if PCT was <1.0 ng/mL or decreased by 80% versus day 1, with resolution of clinical signs. Primary endpoints were time to discontinuation of intravenous antibiotics for the first episode and adverse events. Historical controls were retrieved for propensity score matching. After matching, 30 patients in the PCT group and 60 in the control were included for analysis. The median duration of antibiotic exposure in PCT group was 3.4 days (interquartile range [IQR] 2.2 days), while 6.1 days (IQR 3.2 days) in control (*p* < 0.001). The PCT algorithm significantly improves time to antibiotic discontinuation (*p* < 0.001, log-rank test). The rates of adverse events were comparable between 2 groups. Multivariate-adjusted extended Cox model demonstrated that the PCT-based algorithm was significantly associated with a 87% reduction in hazard of antibiotic exposure within 7 days (hazard ratio [HR] 0.13, 95% CI 0.07–0.21, *p* < 0.001), and a 68% reduction in hazard after 7 days (adjusted HR 0.32, 95% CI 0.11–0.99, *p*  =  0.047). Advanced age, coexisting pulmonary diseases, and higher severity of illness were significantly associated with longer durations of antibiotic use.

**Conclusions/Significance:**

The PCT-based algorithm safely reduces antibiotic exposure in this study. Further randomized trials are needed to confirm our findings and incorporate cost-effectiveness analysis.

**Trial Registration:**

Australian New Zealand Clinical Trials Registry ACTRN12612000601831

## Introduction

Intra-abdominal infection is a common problem in clinical practice, and is the second most common cause of infectious mortality in the intensive care unit [1]. Secondary peritonitis is an intra-abdominal infection that requires urgent intervention and prompt antimicrobial therapy to achieve acceptable outcomes. However, longer durations of antimicrobial therapy are not associated with improved outcomes and may increase the incidence of drug-resistant strains [2]. Recent development of biomarkers such as the procalcitonin (PCT) assay has facilitated antibiotic therapy in several clinical settings [3]–[6].

PCT is the precursor of the hormone calcitonin, and is synthesized physiologically by thyroid C cells [7]. Under normal physiological conditions, serum PCT levels are <0.1 ng/mL; however, systemic PCT secretion has been observed in response to acute inflammation, and it appears to be relatively specific to systemic bacterial infections [8], [9]. Systematic reviews have indicated that PCT is more accurate for diagnosis of bacterial infections than traditional biomarkers such as C-reactive protein [10], [11]. In addition, an observation study demonstrates that concentration of PCT declines from the first postoperative day and reaches half of its initial value by the second day, whereas the mean concentration of C-reactive protein increases in the first 48 hours and reaches half of its maximum value on the fifth day [12]. Thus, since its higher specificity and earlier return to the physiological levels after surgery, PCT should have the ability to help exclude bacterial infections in the early postoperative period.

Several observational studies have demonstrated that PCT level is related to prognosis in secondary peritonitis and its ratio could indicate successful treatment outcomes after abdominal sepsis [13], [14]. Nonetheless, only two randomized trials assessed the efficacy of PCT algorithms for the reduction of durations of antibiotic use versus standard treatment among patients with secondary peritonitis after surgery [15], [16]. Both of them recruited patients admitted to the intensive care unit and had some weaknesses of study designs. Furthermore, recommended durations of antibiotic therapy in complicated intra-abdominal infections following emergency surgery remains inconsistency among evidence-based guidelines [2], [17]. The aims of this study was to investigate whether a PCT-based algorithm could safely reduce the duration of intravenous antibiotic exposure for the first episode among patients with secondary peritonitis after emergency surgery, and provided further information for future randomized trials.

## Materials and Methods

### Study Design and Ethics Statement

This is an investigator-initiated trial enrolling participants between April 2012 and March 2013 at Chang Gung Memorial Hospital, Keelung Branch, in Taiwan. The protocol for this trial and supporting TREND checklist are available as supporting information; see Checklist S1 and Protocol S1. In this prospective non-randomized study, we intended to enroll 30 patients for piloting testing efficacy and safety of the PCT-based algorithm we proposed in our surgical population. If we assumed a mean duration of 7 days in the control group, a standard deviation of 2, 2 days’ reduction by the PCT algorithm (effect size as mean difference), a type I error of 0.05, and power of 80%, we would have needed 16 patients in each group (2 sided t-test with common standard deviation). However, the variance might have been underestimated in previous studies [15], [16]. Additionally, in order to detecting adverse effects such as treatment failures, the sample sizes would be larger. Thus, we decided to include 30 patients in this pilot study. Records of similar types of patients who were treated between January 2010 and December 2011 were retrieved as historical controls for stratification and matching. Our research protocol was approved by the ethical committee of Chang Gung Foundation, and written informed consent was obtained from all participants. In addition, our study protocol had been registered on the Australian New Zealand Clinical Trials Registry in the beginning of this study (https://www.anzctr.org.au/Trial/Registration/TrialReview.aspx?id=362568).

### Inclusion/ Exclusion Criteria

Patients who were at least 20 years old and diagnosed with community-acquired secondary peritonitis with systemic inflammatory response syndrome requiring emergency surgery were eligible for enrollment. Patients who met one or more of the following criteria were excluded: (a) patients who were moribund (life expectancy < 72 hours); (b) patients with advanced liver cirrhosis, Child-Pugh class B or C; (c) patients with profound septic shock under treatment of high-dose inotropic agents; (d) patients with pre-existing infection who received antibiotic treatment; (e) multiple trauma patients with unstable hemodynamic status; (f) pregnant women; (g) immunocompromised patients, i.e., due to human immunodeficiency virus infection, long-term steroid treatment, or chemotherapy; (h) patients or their family declined enrollment.

### Protocol and Intervention

PCT levels were obtained pre-operatively, on post-operative day 1 (24 hours after operation), 3, 5, and 7, and again as needed before stopping antibiotics. PCT levels were also measured after stopping antibiotics, to confirm successful treatment. PCT levels were measured using a rapid sensitive assay with a functional assay sensitivity of 0.06 ng/mL (BRAHMS PCT KRYPTOR assay, Hennigsdorf, Germany). The PCT assay was performed at the central laboratory of our hospital and the results were routinely available within 1 hour. Our algorithm recommended stopping antibiotics if PCT levels decreased by 80% compared to the value on postoperative day 1 or if they were <1.0 ng/mL with resolution of clinical signs including afebrile and tolerance of oral diet.

All patients received the first dose of antibiotics at the emergency department on diagnosis. Two experienced surgeons performed operations for all patients. These surgical procedures included simple closure with omental patch for perforated peptic ulcer, open cholecystectomy for acute cholecystitis, open choledocholithotomy with T-tube drainage for common bile duct stones complicating with acute cholangitis, segmental resection of small bowel with end-to-end anastomosis for small bowel ischemia, and open appendectomy with drainage of intra-abdominal abscess for ruptured appendicitis. After surgery, all participants received empirical antibiotic treatment (ceftriaxone 1 gram intravenous every 12 hours and metronidazole 500 mg intravenous infusion every 8 hours). Infection specialists who were blinded to our PCT algorithm routinely reviewed the justification of antibiotic treatment, including regimens and durations in every patient over the postoperative periods as antibiotic stewardship in our institute. The in-charge physicians decided whether to discontinue antibiotics according to the PCT algorithm, clinical conditions, and suggestions of infection specialists. Owing to the lack of evidence supporting that extension of oral antibiotics improved clinical outcomes, extended use of oral antibiotics were strongly discouraged in this study.

### End points

Our primary endpoints were time to discontinuation of antibiotics (defined from the time of completion of surgery to stopping of antibiotics for the first episode) and adverse events. Death from any cause was regarded as a serious adverse event. Intensive care unit re-admission for any reason, disease-specific complications (i.e., persistence or development of intra-abdominal infections, re-operation), and deep surgical site infections were defined as adverse events.

### Statistical analysis

To reduce selection bias and confounding bias, our analysis planning included propensity score matching, stratification, and regression analysis [18], [19]. We conducted the propensity score matching stratified by disease etiologies (hollow organ perforation, acute cholecystitis, acute cholangitis, ruptured appendicitis, and bowel ischemia) with a 1∶2 ratio. We included age, total number of comorbidities (cardiovascular disease, cerebrovascular disease, diabetes mellitus, renal dysfunction, and pulmonary disease), leukocyte counts, Acute Physiological and Chronic Health Evaluation (APACHE II) score [20] (≥15 or <15), and Mannheim peritonitis index [21] (MPI) as our propensity score model. The analyst was blinded to the outcome data at this stage. Continuous variables were expressed as median with interquartile range (IQR), whereas categorical variables were presented as frequency and percentage. Because our primary endpoint was time to discontinuation of antibiotics, we constructed Kaplan-Meier survival curves stratified by treatment. In univariate analysis, baseline characteristics, occurrences of adverse events, and the time to discontinuation of antibiotics (in days) were compared between the PCT group and matched controls using chi-squared test, Fisher’s exact test, Wilcox rank-sum test, or log-rank test as appropriate. Next, we conducted multivariate analysis using the Cox proportional hazards model. The stepwise variable selection procedure (with iterations between forward and backward steps) was applied to obtain the candidate final regression model. Moreover, we applied statistical tools for regression diagnostics, such as the check for proportional hazards assumption, residual analysis, detection of influential cases, and the check for multicollinearity, to uncover problems of the model or data. For the violation of proportional hazards assumption, we used the extended Cox model with time-varying covariates to conduct multivariate analysis [22]. In addition, R2 values were examined to assess the goodness-of-fit of the fitted Cox model. All reported confidence intervals (CIs) and tests were 2-sided with a 5% significance level. All analyses were performed with R version 3.0.1 (R Foundation for Statistical Computing, Vienna, Austria). Propensity score matching was performed with the contributed R package “MatchIt” [23]–[25], and survival analysis was conducted with the “survival” package. Additionally, a post hoc power assessment in our study showed a power of 80% to detect a 50% reduction in hazards in the PCT group, assuming proportional hazards and adjusting for APACHE II scores at an alpha level of 0.05. The power calculation was conducted with contributed R package “powerSurvEpi” [26].

## Results

**Figure 1:**
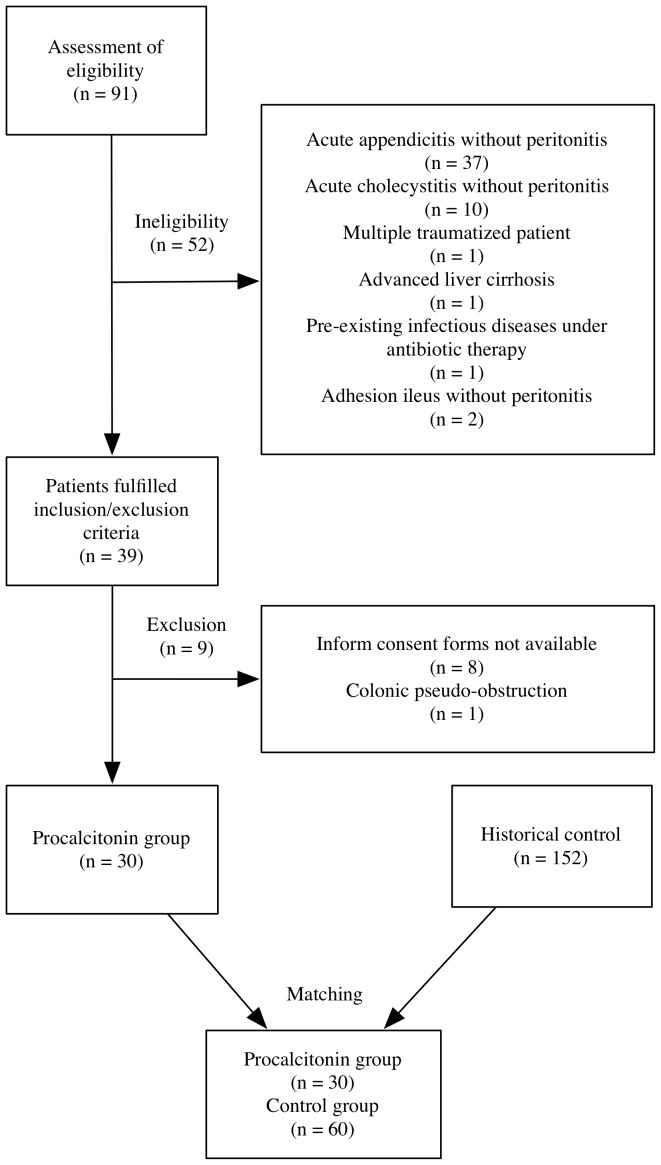
Flow diagram of the study.

From April 2012 to March 2013, 30 patients fulfilling inclusion/exclusion criteria participated in this prospective clinical trial (Figure 1). There were 15 patients with hollow organ perforation, 5 patients with ruptured appendicitis, 4 patients with small bowel ischemia, 3 patients with acute cholecystitis, and 3 patients with acute cholangitis. Moreover, we identified data from records of 152 similar patients treated between January 2010 and December 2011 as the control group. After stratifying and matching, we included 30 patients in the PCT group, whereas 60 in control.

The baseline characteristics of the matched groups are shown in Table 1. There were no statistically significant differences of baseline characteristics between 2 groups, and the matching balance of treatment and control groups improved for age, comorbidities, disease etiologies, and APACHE II score (Table 1 and Table S1). The majority of patients in both groups had hollow organ perforation. There were statistically significant differences between 2 groups for the median duration of intravenous antibiotic therapy. The rates of adverse events were comparable between 2 groups.

**Table 1:** Characteristics and outcomes of the matched cohorts.

Characteristics	PCT group		Control group		P value
	(n = 30)		(n = 60)		
**Age, y** *	70	(34.0)	67	(28.5)	0.811
**Male sex, no. (%)**	18	(60.0)	37	(61.7%)	0.878
**Coexisting illnesses, no. (%)**					
Cardiovascular disease	10	(33.3)	13	(21.7)	0.232
Pulmonary disease	4	(13.3)	13	(21.7)	0.405
Cerebrovascular disease	3	(10.0)	7	(11.7)	1
Renal dysfunction	6	(20.0)	10	(16.7)	0.772
Diabetes mellitus	9	(30.0)	16	(26.7)	0.739
Malignancy	4	(13.3)	8	(13.3)	1
**Disease etiology, no. (%)**					
Hollow organ perforation	15	(50.0)	30	(50.0)	1
Acute cholecystitis	3	(10.0)	6	(10.0)	
Acute cholangitis	3	(10.0)	6	(10.0)	
Ruptured appendicitis	5	(16.7)	10	(16.7)	
Bowel ischemia	4	(13.3)	8	(13.3)	
**Laboratory findings**					
Preoperative leukocyte count, cells/µL *	11850	(8525)	11900	(7000)	0.844
**Severity scores**					
Mannheim peritonitis index *	23	(5.75)	22	(6)	0.823
APACHE II ≥ 15, no. (%)	8	(26.7)	10	(16.7)	0.264
**Morbidity, no. (%)**					
Any adverse outcomes	11	(36.7)	16	(26.7)	0.329
Deep SSI/organ space SSI	3	(10.0)	5	(8.3)	1
Medical complications	7	(23.3)	10	(16.7)	0.446
**Mortality, no. (%)**	0	(0.00)	6	(10.0)	0.173
**Antibiotics use**					
Intravenous antibiotic use, d *	3.4	(2.2)	6.1	(3.2)	<0.001
Extended oral antibiotic use, no. (%)	1	(3.3)	20	(33.3)	0.001
**Propensity scores** *	0.28	(0.30)	0.20	(0.16)	0.077

*Data are expressed as Median (IQR; interquartile range).

APACHE II, Acute Physiological and Chronic Health Evaluation score; PCT, procalcitonin; SSI, surgical site infection.

Independent variables in propensity score models: age, number of comorbidities, preoperative leukocyte count, APACHE II score, and Mannheim peritonitis index.

The dependent variable in propensity score models: treatment type (PCT group or Control group).

Propensity scores represent the probability of patients receiving PCT or control group treatment given independent variables in propensity score models.

**Figure 2:**
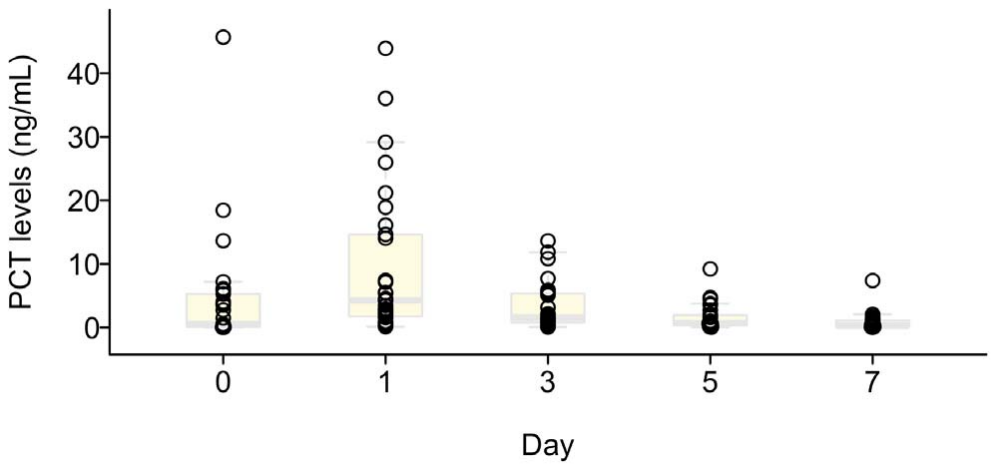
Box plot of procalcitonin concentrations during pre-operative and post-operative periods. The majority of day 1 procalcitonin levels declined nearly to physiological levels within 3 to 5 days. PCT, procalcitonin.

**Figure 3:**
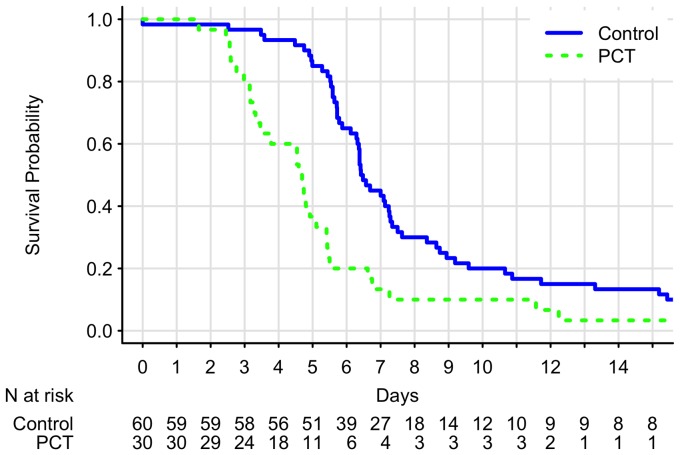
Kaplan-Meier survival curves. The results demonstrate that time to antibiotic discontinuation significantly improves in the treatment group (*p* < 0.001, log-rank test). The majority of patients in the control group discontinued antibiotics between postoperative day 4 and day 8. PCT, procalcitonin.

Figure 2 shows PCT levels of the treatment group. The PCT levels decreased to <1.0 ng/mL within 3–5 days among the majority of patients. Figure 3 demonstrates the Kaplan-Meier survival curves stratified by treatment. There was statistically significant difference between 2 groups (*p* < 0.001, log-rank test). Eight patients in the control group and 1 patient in the PCT group developed infectious complications that required prolonged antibiotic treatment during the postoperative recovery period. In sensitivity analysis, when we truncated at 14 days for these 9 patients and treated them as censorship, the result was still highly statistically significant (*p* < 0.001, log-rank test).

The results of multivariate Cox proportional hazards model indicated that treatment type, advanced age, the presence of pulmonary comorbidities, and APACHE II score were statistically significant associated with durations of post-operative intravenous antibiotic exposure in the final model. However, the graph of the log (−log [survival probability]) versus the log of survival time suggests that the proportional hazards assumption dose not hold (Figure S1) [22]. Also, the statistical approach by testing correlations between Schoenfeld’s residuals and ranked failure times shows that the proportional hazards assumption is violated for the treatment covariate (Table S2) [22]. Both of them indicate that the hazard ratio for treatment effects is not a constant during the postoperative period. Therefore, we performed the multivariate-adjusted extended Cox model with time-dependent variables for treatment effects. Table 2 summarizes the results of the extended Cox model. After adjustment for confounding, the PCT-based algorithm was significantly associated with a 87% reduction in hazard of postoperative antibiotic exposure within 7 days (hazard ratio [HR] 0.13, 95% CI 0.07–0.21, *p* < 0.001), and a 68% reduction in hazard after 7 days (adjusted HR 0.32, 95% CI 0.11–0.99, *p*  =  0.047). Advanced age, coexisting pulmonary diseases, and higher severity of illness (APACHE II ≥ 15) were significantly associated with longer durations of antibiotics use. The choice of day 7 as the cut-off point was mainly based on inputs from clinical practice. Evidence-based guidelines recommend that antibiotic treatment should be limited to 4–7 days during the postoperative period. Clinically, most patients could receive less than 7 days of antibiotic therapy, unless postoperative infectious complications occur. When we tried day 5 as the cut-off value, the results were not changed (data not shown).

**Table 2:** Results of the multivariate-adjusted Cox model with time-dependent variables.

	HR*	(95% CI)	*P* value
**Postoperative period <7 days**			
Control group	1	[Reference]	
PCT group	0.13	(0.08–0.22)	<0.001
**Postoperative period ≥ 7 days**			
Control group	1	[Reference]	
PCT group	0.32	(0.11–0.99)	0.047
**Pulmonary diseases**			
No	1	[Reference]	
Yes	2.38	(1.23–4.59)	0.010
**APACHE II score**			
<15	1	[Reference]	
≥15	3.84	(1.74–8.50)	<0.001
**Age, per year**	1.02	(1.01–1.03)	0.004
**Propensity score**	1.66	(0.36–7.55)	0.513

^*^ We calculated the reciprocal values of exponential coefficients from an extended Cox model to facilitate interpretations.

Goodness-of-fit test: *R* ^2^  =  0.434.

APACHE II, Acute Physiological and Chronic Health Evaluation score; HR, hazard ratio; CI, confidence interval; PCT, procalcitonin.

### Adherence with Study Algorithm and Guidelines

Our protocol adherence in the PCT group according to the PCT criteria was 81.5%. In the PCT group, 7 patients (25.9%) had PCT concentrations <1.0 µg/L on postoperative day 1. Five of 7 patients received extended intravenous antibiotic therapy due to coexisting illness and recommendations of infection specialists. Among these 5 patients, 4 had treatment extended by 2 days, and 1 had a 4-day treatment extension. None of these 5 patients developed further complications. In the control group, antibiotics were administered for 4 to 8 days among the majority of the patients indicating acceptable adherence to the evidence-based guidelines (Figure 3).

### Microbiological Findings and Treatment Failures

We summarize the microbiological findings and treatment failures as well as the antibiotic coverage information of our empiric regimen in Table 3. The number of identified strains per patient was comparable in the two groups. The median duration of antibiotic treatment in the subgroup with positive cultures was 5 days in the PCT group, whereas it was 7 days in the control group. Of note, there were two patients (one case of peptic ulcer perforation and one case of bowel ischemia) in the PCT group that developed a urinary tract infection and pneumonia resulting from treatment failures that needed additional antibiotic treatment. Treatment failure was defined if cultures obtained after treatment showed microbiological organisms that were the same as those in previous ascites culture reports. On the other hand, there were two patients (one case of peptic ulcer perforation and one case of bowel ischemia) in the control group diagnosed with pneumonia that also needed additional antibiotic treatment. One of the patients died of multiple organ failure.

**Table 3:** Microbiological and antibiotic treatment information from the two groups.

	PCT group (n = 30)	Control group (n = 60)
**No. of organisms identified per patient**		
Zero growth, no. (%)	12 (40)	29 (48.3)
1 strain, no. (%)	6 (20)	13 (21.7)
2 strains, no. (%)	3 (10)	6 (10)
3 strains, no. (%)	3 (10)	7 (11.7)
4 strains, no. (%)	1 (3.3)	2 (3.3)
5 strains, no. (%)	5 (16.7)	2 (3.3)
6 strains, no. (%)	0 (0)	1 (1.7)
**Antibiotic duration with positive culture reports, d, median (IQR)**	5 (1.5)	7 (6.8)
**Patients with strains resistant to empirical regimens, no. (%)**	8 (26.7)	12 (20)
**Types of treatment failure (strain)**	Urinary tract infection (*Pseudomonas aeruginosa*),	Pneumonia (*Pseudomonas aeruginosa*), Pneumonia (*Enterococcus faecalis*)
	Pneumonia (*Escherichia coli*-ESBL strain)	

ESBL, extended-spectrum β-lactamase; IQR, interquartile range, PCT, procalcitonin.

## Discussion

Our prospective study indicated that the PCT-guided algorithm, compared to the standard of care, significantly reduced the hazard of antibiotic exposure among patients with secondary peritonitis following emergency surgery. In addition, it does not increase the risk of adverse events. Advanced age, coexisting pulmonary diseases, and higher severity of illness (APACHE II score ≥15) were significantly associated with longer durations of antibiotic administration.

Concerns regarding the use of surrogate markers in terminating antibiotic treatment are related to the possibility that treatment failure might occur, particularly in patients with difficult-to-treat organisms such as *Pseudomonas aeruginosa* or strains with extended-spectrum β-lactamase. Indeed, our study demonstrates that empiric antibiotic regimens for patients with secondary peritonitis caused by difficult-to-treat organisms are at a high risk for treatment failure. These patients will require additional courses of antibiotic treatment if treatment failure occurs, and might develop unfavorable outcomes. In addition, given the brief treatment duration of patients managed by the PCT protocol, it is not likely that regimens would be changed based on the findings of microbiological studies. Future studies should take these issues into account during the trial design stage.

For adult patients with complicated intra-abdominal infections, current evidence-based guidelines recommend prophylactic antibiotics within 24 hours for acute gastric and proximal perforations and limited antimicrobial therapy to 4–7 days unless it is difficult to achieve source control [2]. These recommendations are mainly based on opinions of respected authorities, clinical experience, descriptive studies, and reports of expert committees. Scanty prospective randomized trials investigated durations of antibiotic use among patients with secondary peritonitis after emergency surgery. In addition, valid risk stratification models in order to allocate low-risk patients for earlier discontinuation of antibiotics are not yet available. Therefore, guidelines have recommended that there is a pressing need for the establishment of appropriate durations of antimicrobial therapies in this population [2]. The present study provides some timely evidence for such recommendations and the use of PCT-based algorithm may be more specific to individual patient.

To the best of our knowledge, only 2 randomized controlled trials assessing the efficacy of PCT algorithms to guide antibiotic treatment among surgical patients have been published so far [15], [16]. There are several discrepancies between our study and theirs. First, Schroeder and colleagues recruited only 19 patients with peritonitis after abdominal surgery among a total of 27 patients in 2 groups [15], whereas Hochreiter and colleagues enrolled patients with confirmed or highly suspected bacterial infections requiring antibiotic treatment in the surgical intensive care setting [16]. Second, both of them discontinued antibiotics if there was clinical improvement observed and PCT level was <1.0 ng/mL or decreased to 25% to 35% of its initial value for 3 days according to daily PCT levels. In addition, Hochreiter and colleagues prescribed 8-day antibiotic treatment for all patients in the control group. Such treatment strategy compromised generalizability in clinical settings, and might overestimate the treatment effect. Third, both of them adopted treatment durations (in days) as their outcome measures. The use of continuous variables as outcome measures in such studies would introduce bias if infectious complications requiring antibiotic therapies happened, and the sample size is small. Therefore, our study is different from theirs with regard to research questions and study designs.

In patients with respiratory tract infections, PCT algorithms are associated with 3–4 fewer days of antibiotic exposure without increases in mortality from all causes or treatment failures [3]. A systematic review showed that the percentage of relative reduction of antibiotic duration ranged from 13–55% for different infections [5]. In our study, the PCT algorithm reduced antibiotic use by 3 days and offered a 50% relative reduction in antibiotic duration. Together with the relative reduction in the hazards of antibiotic exposure, the current study provides the treatment effects of PCT algorithms for conducting future randomized trials.

Clinically, it is reasonable that the treatment effect of the PCT-based algorithm is not a constant during the postoperative period. Most of patients should receive postoperative antibiotic treatment less than 7 days for the episode of secondary peritonitis unless clinically needed. The antibiotic administration more than 7 days may be governed by occurrences of postoperative infectious complications or inability to achieve adequate source control. In addition to treatment effects, our findings suggested that advanced age, pulmonary comorbidities, and higher severity of illness are significantly associated with longer durations of antibiotics exposure. These are consistent with previous studies that advanced age, hypoalbuminemia, malnutrition, comorbidities, and higher severity of illness (APACHE II score ≥15) have been reported at risk for the failure of source control and development of infectious complications [2], [27]–[29].

Our study has several advantages. First, time to event outcome is more plausible than continuous outcome in this kind of study. Such approach would allow us to investigate time-varying treatment effects and adjusting for competing risks (when mortality is a competing risk for earlier discontinuation of antibiotics). In this study, only one patient died within 7 days in the control group. We did not conduct a competing risk analysis because of the very low occurrence of this event (1/60). Second, trials of PCT algorithms cannot be blinded and are susceptible to performance bias. Thus, if selection bias could be minimized, the results of well-conducted nonrandomized studies would be comparable to randomized trials. Propensity score matching has been widely adopted when making causal inferences in non-randomized experimental trials. Nevertheless, our study has several limitations. First, unmeasured confounding factors could not be specified in the propensity score models. Second, performance bias exists due to the lack of blinding. Such bias would lead to overestimate the treatment effect. Third, information bias is a concern for the use of historical controls in our study. Fourth, given the use of historical controls, our study might have overestimated the treatment effect. Fifth, owing to potential insufficient statistical power of the present study, a further large controlled clinical trial is needed to investigate the safety endpoints using non-inferiority tests. Finally, owing to the small sample size, our study could not have adequate power to detect some potential confounding such as hypoalbuminemia, malnutrition, and MPI.

## Conclusions

In summary, our study provides some timely evidence for evidence-based guidelines recommending short-term postoperative antibiotic therapy in this population. In addition, our report offers valuable information for future randomized trials including study designs and estimated effect sizes. Moreover, future studies are encouraged to investigate cost-effectiveness by incorporating costs of PCT assays and potential savings in consumption of antibiotics and other healthcare resources, as well as the secondary cost savings due to lower risk of side effects of antibiotics and decreased drug-resistant strains.

## Supporting Information

Figure S1 **The log-log survival curves versus survival time.** The result suggests the proportional hazard assumption does not hold after day 7. The hazard ratio is not a constant in this situation.
(TIF)

Table S1 **Baseline characteristics of the unmatched study cohorts.**
(DOC)

Table S2 **Statistical approach testing the proportional hazards assumption.**
(DOC)

Protocol S1 **Study protocol.**
(DOCX)

Checklist S1 **TREND checklist.**
(DOCX)
