# Supplementary figures and images for: A Procalcitonin-Based Algorithm to Guide Antibiotic Therapy in Secondary Peritonitis following Emergency Surgery: A Prospective Study with Propensity Score Matching Analysis
Source: PLoS One. 2014 Mar 4;9(3):e90539. doi: 10.1371/journal.pone.0090539 (PMC3942439; doi:10.1371/journal.pone.0090539)

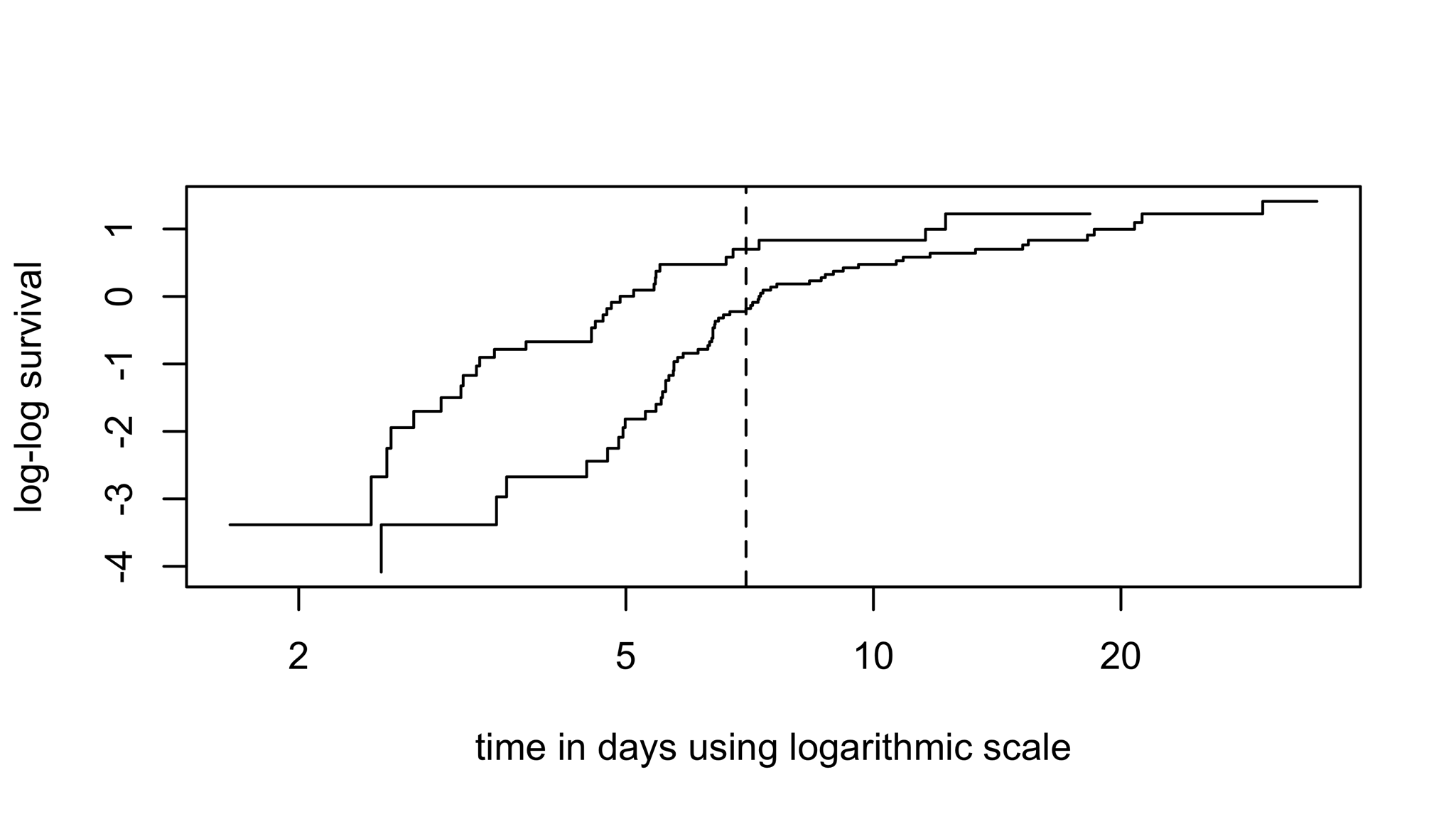

Supplement: Figure S1 — The log-log survival curves versus survival time. The result suggests the proportional hazard assumption does not hold after day 7. The hazard ratio is not a constant in this situation. (TIF) [file pone.0090539.s001.tif]
