# Supplementary material for: A Procalcitonin-Based Algorithm to Guide Antibiotic Therapy in Secondary Peritonitis following Emergency Surgery: A Prospective Study with Propensity Score Matching Analysis
Source: PLoS One. 2014 Mar 4;9(3):e90539. doi: 10.1371/journal.pone.0090539 (PMC3942439; doi:10.1371/journal.pone.0090539)
Supplement: Table S1 — Baseline characteristics of the unmatched study cohorts. (DOC) [file pone.0090539.s002.doc]

**Table S1** Baseline characteristics of the unmatched study cohorts

| **Characteristics** | **PCT group** | | **Control group** | | **P value** |
| --- | --- | --- | --- | --- | --- |
|  | (n = 30) | | (n = 152) | |  |
| **Age, y *** | 70 | (34.0) | 74 | (20.5) | 0.162 |
| **Male, no. (%)** | 18 | (60.0) | 93 | (61.2) | 0.903 |
| **Coexisting illnesses, no. (%)** |  |  |  |  |  |
| Cardiovascular disease | 10 | (33.3) | 32 | (21.2) | 0.145 |
| Pulmonary disease | 4 | (13.3) | 38 | (25.0) | 0.166 |
| Cerebrovascular disease | 3 | (10.0) | 22 | (14.5) | 0.772 |
| Renal dysfunction | 6 | (20.0) | 30 | (19.7) | 0.974 |
| Diabetes mellitus | 9 | (30.0) | 35 | (23.0) | 0.415 |
| Malignancy | 4 | (13.3) | 15 | (9.9) | 0.524 |
| **Disease etiology, no. (%)** |  |  |  |  |  |
| Hollow organ perforation | 15 | (50.0) | 64 | (42.1) | 0.119 |
| Acute cholecystitis | 3 | (10.0) | 31 | (20.4) |  |
| Acute cholangitis | 3 | (10.0) | 34 | (22.4) |  |
| Ruptured appendicitis | 5 | (16.7) | 14 | (9.2) |  |
| Bowel ischemia | 4 | (13.3) | 9 | (5.9) |  |
| **Laboratory findings** |  |  |  |  |  |
| Preoperative leukocyte count, cells/μL* | 11850 | (8525) | 12400 | (9500) | 0.681 |
| **Severity scores** |  |  |  |  |  |
| Mannheim peritonitis index (MPI) * | 23 | (5.75) | 21 | (6.00) | 0.407 |
| APACHE II ≥15, no. (%) | 8 | (26.7) | 14 | (9.2) | 0.013 |

*APACHE II*, Acute Physiological and Chronic Health Evaluation score; *PCT*, procalcitonin. *Data are expressed as Median (IQR; interquartile range)
