# Supplementary material for: A Procalcitonin-Based Algorithm to Guide Antibiotic Therapy in Secondary Peritonitis following Emergency Surgery: A Prospective Study with Propensity Score Matching Analysis
Source: PLoS One. 2014 Mar 4;9(3):e90539. doi: 10.1371/journal.pone.0090539 (PMC3942439; doi:10.1371/journal.pone.0090539)
Supplement: Table S2 — Statistical approach testing the proportional hazards assumption. (DOC) [file pone.0090539.s003.doc]

**Table S2.** Statistical approach testing the proportional hazards assumption*

|  | **rho** | **Chi-square** | ***p* value** |
| --- | --- | --- | --- |
| **Treatment** | -0.249 | -5.736 | 0.017 |
| **Age** | -0.197 | 3.384 | 0.066 |
| **Comorbidities (no.)** | 0.139 | 1.907 | 0.167 |
| **Pulmonary disease** | -0.037 | 0.142 | 0.706 |
| **APACHE II ≥15** | 0.126 | 1.755 | 0.185 |
| **GLOBAL** | NA | 10.975 | 0.052 |

*This method included Schoenfeld residuals for each predictor using a chi-square statistic with 1 degree of freedom and correlations between Schoenfeld’s residuals and ranked failure times. If the *p* value is significant, then the proportional hazards assumption is violated.

*APACHE II*: Acute Physiological and Chronic Health Evaluation score.
