# Supplementary material for: A Procalcitonin-Based Algorithm to Guide Antibiotic Therapy in Secondary Peritonitis following Emergency Surgery: A Prospective Study with Propensity Score Matching Analysis
Source: PLoS One. 2014 Mar 4;9(3):e90539. doi: 10.1371/journal.pone.0090539 (PMC3942439; doi:10.1371/journal.pone.0090539)
Supplement: Protocol S1 — Study protocol. (DOCX) [file pone.0090539.s004.docx]

**Protocol S1**

**A pilot study of procalcitonin-based algorithms to guide antibiotics treatment in secondary peritonitis after surgery**

**血清降鈣素原指引在腹膜炎術後抗生素使用的先期試驗**

**PI:**

黃挺碩 (Ting-Shuo Huang)

**Co-PIs:**

陳晃洋 (Huang-Yang Chen)

黃協賢 (Shie-Shian Huan)

許邦弘 (Pang-Hung Hsu)

**Biostatistician:**

胡賦強 (Fu-Chang Hu)

**Abstract in Chinese**

腹膜炎是腹腔內感染造成敗血症最主要的原因，而且常導致嚴重的併發症甚至死亡。目前仍然缺乏足夠的實証醫學證據告訴我們，這一類病人術後抗生素需要施打多久。過長的抗生素治療會造成多重抗藥性菌種的產生、抗生素的副作用及醫療費用。系統性回顧顯示出，血清降鈣素原比Ｃ反應蛋白在鑑別細菌性和非細菌性所造成的感染有更優越的敏感性和專一性。血清降鈣素原可以增加重症病患敗血症診斷的準確度和社區性肺炎診斷的準確度。在兩個大型的隨機對照試驗指出，在下呼吸道感染的病人，血清降鈣素原指引和傳統的標準作法比較起來，有相近的預後但是暴露在抗生素使用的天數及抗生素所造成的不良反應卻顯著的減少。另外在一些小型的研究指出，血清降鈣素原指引可減少外科重症病患術後抗生素的使用天數。日前在亞洲甚至台灣還沒有相關的研究。因此，我們設計這個先期試驗主要探討血清降鈣素原指引是否可以安全的、有效的在腹膜炎需要手術治療的病患，減少術後抗生素的使用。我們預計會在一年的時間內收集三十個病患，會和過去兩年同疾病的病人做比較。此外，我們將會用質譜分析探索新的敗血症生化標記，以期在未來對於疾病的診斷、預後及治療方面有更進一步的進展。

**Abstract in English**

Peritonitis is one of the most important etiologies resulting in abdominal sepsis leading to considerable morbidity and mortality. However, there is still no adequate evidence-based data about the optimal duration of antibiotics (AB) treatment for moderate to severe complicated intra-abdominal infections after abdominal surgery. Prolonged duration of AB therapy can increase the risk of developing multidrug-resistant strains as well as adverse effects of AB and treatment associated costs. A systematic review showed that procalcitonin (PCT) levels were more sensitive and more specific than C-reactive protein (CRP) levels for differentiating bacterial from non-infective causes of inflammation. PCT improves the clinical diagnosis of sepsis among critically-ill patients and patients with community-acquired pneumonia. Among patients with lower respiratory tract infections, a strategy of PCT guidance AB therapy compared with standard guidelines resulted in similar rates of adverse outcomes, as well as lower rates of antibiotic exposure and AB-associated adverse effects. Some small studies in surgical intensive unit demonstrated that PCT-based algorithms might reduce AB exposure without compromising clinical outcomes. We design this pilot study to investigate the feasibility and safety of PCT-based algorithms to guide post-operative AB therapy in secondary peritonitis. We will prospectively enroll 30 patients in this study for one-year period. Appropriate historical control of the same kind of patients will be collected from January 2010 to December 2012. To investigate predictive and prognostic value of novel biomarkers, we will also explore the potential biomarkers of sepsis. In this study, we expect to shorten and optimize the duration of AB therapy in secondary peritonitis after surgery without compromising clinical outcomes, provide more information about novel biomarkers, and provide standardized proteomic methods to detect them.

**Background and Rationale**

Peritonitis is one of the most important etiologies resulting in abdominal sepsis leading to considerable morbidity and mortality. Secondary peritonitis usually requires urgent surgical intervention to treat intra-abdominal infections (IAI). However, there is still no adequate evidence-based data about the optimal duration of antibiotics (AB) treatment for moderate to severe complicated IAI after abdominal surgery. The duration of AB treatment in patients with secondary peritonitis is based on empirical or guideline-based considerations. Therefore, the recommended duration of AB therapy in IAI is still controversial and no consensus has been reached due to lack of well-designed controlled studies that would provide sufficient scientific evidence. Excessive duration of antibiotic therapy can increase the risk of developing multidrug-resistant strains as well as adverse effects and treatment associated costs. These considerations have led to the exploration of "short-term treatment" strategies, lasting 4-7 days, in recent published guidelines by the Surgical Infection Society and the Infectious Diseases Society of America (IDSA) [[1](#_ENREF_1)]. In Taiwan, our guidelines developed by Infectious Diseases Society of Taiwan (IDST) suggested 7-10 days AB treatment in local peritonitis and 10-14 days antibiotics treatment for generalized peritonitis. For high-risk patients, second-line AB should be first considered [[2](#_ENREF_2), [3](#_ENREF_3)].

World Health Organization (WHO) calls for urgent and concerted action to slow down the spread of drug resistance on 07 April 2011. In fact, the issue of drug resistance is becoming more and more important because many infectious diseases are no longer easily cured such as pulmonary tuberculosis and health care-associated resistant strains infections. Recent development of biomarkers opens the door to personalized medicine that might allow the duration of AB treatment in intra-abdominal and other infections to be tailed to the response of individual patient. Among biomarkers, C-reactive protein (CRP) is the most well-known and widely used. CRP is synthesized principally by hepatocytes in response to stimulation by cytokines, and its plasma half-life is about 19 hours. In healthy adults, the normal plasma concentration of CRP is about 0.8 mg/L [[4](#_ENREF_4), [5](#_ENREF_5)]. A useful biomarker of infection should provide important information to the clinical practice including diagnosis, disease severity and prognosis, and therapeutic guidance [[6](#_ENREF_6)]. The key disadvantages of CRP are not specific for sepsis and that its levels may be increased in other inflammatory and surgical conditions.

Procalcitonin (PCT) is the precursor of hormone calcitonin (CT), and is synthesized physiologically by thyroid C cells [[7](#_ENREF_7)]. In normal physiological situations, PCT levels in the serum are very low (<0.1 ng/mL). However, PCT is synthesized in various extrathyroidal parenchymal tissues such as adipocytes, liver, lung, brain, and muscles in bacterial infection. Systemic PCT secretion is a response of the acute inflammation that appears to be relatively specific to systemic bacterial infections. PCT improves the clinical diagnosis of sepsis in critically-ill patients and patients with community-acquired pneumonia [[8](#_ENREF_8), [9](#_ENREF_9)]. Two large randomized controlled trials demonstrated that PCT is a promising candidate for AB stewardship in lower respiratory tract infections (LRTI) [[10](#_ENREF_10), [11](#_ENREF_11)]. In patients with LRTIs, a strategy of PCT guidance AB therapy compared with standard guidelines resulted in similar rates of adverse outcomes, as well as lower rates of antibiotic exposure and antibiotic-associated adverse effects.

We conduct comprehensive reviews of evidence-based databases using PICO (patient/problem, intervention, comparison, outcomes) formats (Table 1). Our search strategies are using meta-search engines including Trip database ([www.tripdatabase.com](http://www.tripdatabase.com), Fig. 1) and SUMSearch 2 (sumsearch.org, Fig. 2) suggested by the Centre for Evidence Based Medicine ([www.cebm.net](http://www.cebm.net)). The results were summarized in Table 2-6.

Table 1. PICO formats

| **P** | Peritonitis, sepsis, bacterial infection | Peritonitis, sepsis, bacterial infection |
| --- | --- | --- |
| **I** | Procalcitonin | PCT-guided algorithyms |
| **C** |  | EBM guidelines |
| **O** | Diagnostic accuracy | Benefit: shorter duration of AB exposure, less adverse events  Harm: treatment failure |

Fig 1. Trip database ([www.tripdatabase.com](http://www.tripdatabase.com)) Accessed date: 2011/12/18


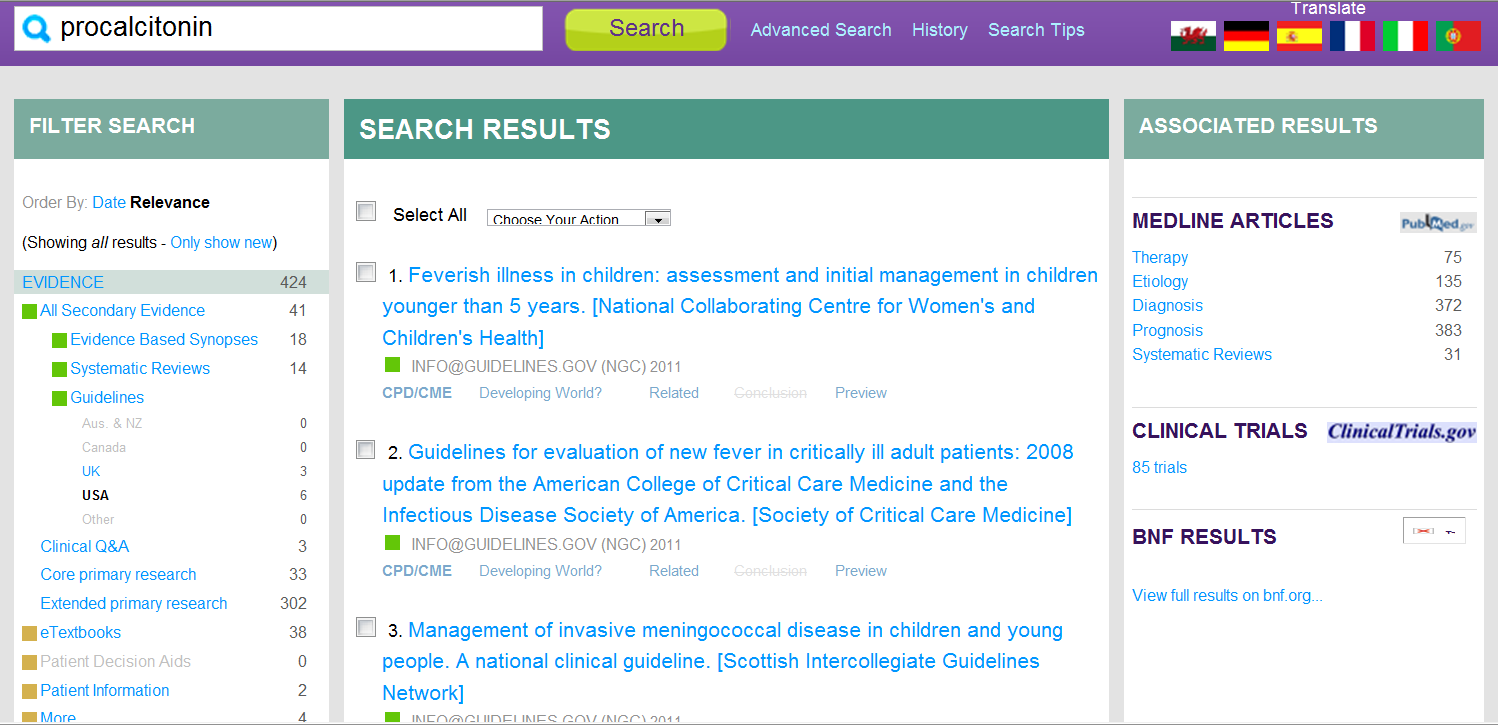


Table 2. Results of all secondary evidence

| All secondary database | 41 | Diagnostic marker | Therapeutic guidance | Not relevant | RCT |
| --- | --- | --- | --- | --- | --- |
| Systematic reviews | 14 | 1 (DARE)  1 (HTA) | 3 (DARE) | 8 | 1 |
| Evidence based synopses | 18 | 1 (duplicate) | 0 | 15 | 2 |
| Guidelines | 9 | 1 (NGC) | 0 | 8 | NA |

Database of Abstracts of Reviews of Effects ( DARE ), Health Technology Assessment (HTA)

National guideline clearinghouse (NGC), Randomized controlled trial (RCT)

Table 3. Results of PubMed search

| PubMed | 31 |
| --- | --- |
| Therapeutic guidance | 6 (3 duplicate) |
| Diagnostic marker | 3 (1 duplicate) |

Table 4. Results of SUMSearch 2

| SUMSearch2 (SR) | Not relevant | Duplicate |
| --- | --- | --- |
| 30 | 24 | 6 |

Systematic review (SR), NGC guidelines=6 (duplicated with Trip database)

Fig 2. SUMSearch 2 (sumsearch.org) Accessed date: 2011/12/18


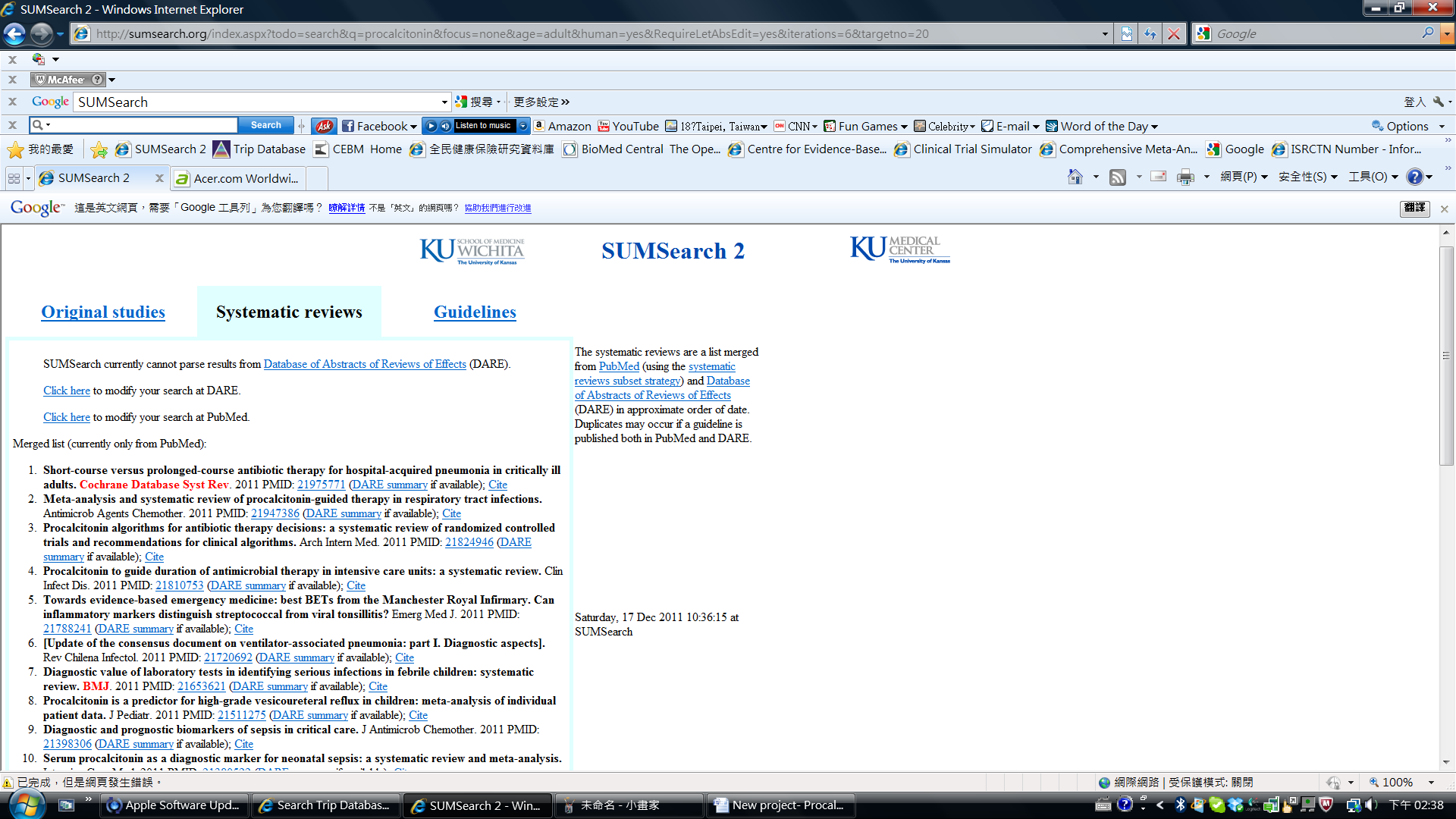


Table 5. PCT as a diagnostic marker

| Year | Author | Title | Journal | Comment |
| --- | --- | --- | --- | --- |
| 2007 | Jones A E et al. | Procalcitonin test in the diagnosis of bacteremia: a meta-analysis. | Annals of Emergency Medicine | Low quality, Heterogeneity, Publication bias, Assay methods not reported, Trip-database |
| 2007 |  | Serum procalcitonin as a marker of bacterial lower respiratory tract infection | National Horizon Scanning Centre News | Trip-database |
| 2007 | Benjamin M P Tang et al. | Accuracy of procalcitonin for sepsis diagnosis in critically ill patients: systematic review and meta-analysis | Lancet Infect Dis | PubMed |
| 2006 | Bernard Uzzan et al. | Procalcitonin as a diagnostic test for sepsis in critically ill adults and after surgery or trauma: A systematic review and meta-analysis | Crit Care Med | PubMed |

Table 6. PCT as a therapeutic guidance

| Year | Author | Title | Journal | Comment |
| --- | --- | --- | --- | --- |
| 2011 | Schuetz P et al. | Procalcitonin algorithms for antibiotic therapy decisions: a systematic review of randomized controlled trials and recommendations for clinical algorithms. | Archives of Internal Medicine | High quality, Trip-database |
| 2010 | Kopterides P et al. | Procalcitonin-guided algorithms of antibiotic therapy in the intensive care unit: a systematic review and meta-analysis of randomized controlled trials | Critical Care Medicine | Low quality, Publication bias, Heterogeneous primary outcomes,  Trip-database |
| 2009 | Tang H et al. | Effect of procalcitonin-guided treatment in patients with infections: a systematic review and meta-analysis | Infection | Low quality, Heterogeneity, Indirectness, Trip-database |
| 2011 | Rajender Agarwal et al. | Procalcitonin to Guide Duration of Antimicrobial Therapy in Intensive Care Units: A Systematic Review | Clinical Infectious Disease | PubMed |
| 2011 | Hui Li et al. | Meta-Analysis and Systematic Review of Procalcitonin-Guided Therapy in Respiratory Tract Infections | Antimicrobial Agents and Chemotherapy | PubMed |
| 2011 | Daren K. Heyland et al. | Procalcitonin for reduced antibiotic exposure in the critical care setting: A systematic review and an economic evaluation | Crit Care Med | PubMed |

In summary, single biomarker is not likely promisingly to detect sepsis. Multi-panel biomarkers are the direction of future research. PCT-guided antibiotic treatment is likely to shorten the antibiotics exposure in lower respiratory tract infection by 2 days. But in critically ill septic patients, further research is needed before the PCT test can be recommended for widespread clinical use due to heterogeneous study populations and strategies. The available evidence suggested that the costs of an additional diagnostic tests might be balanced by savings from reduced antibiotic prescriptions. Here we propose this pilot study to test PCT-based algorithms to guide postoperative antibiotics treatment in secondary peritonitis before we conduct large-scale randomized controlled trials due to heterogeneous PCT-guided criteria in different studies. In addition, we will explore other potential biomarkers using proteomic approaches to aid the performance of diagnoses, predict prognosis, and guide clinical practice in future studies [[12-14](#_ENREF_12)].

**Objectives and Specific Aims**

*The research question/hypothesis: Does PCT-based algorithms to guide antibiotic therapy reduce the risk of antibiotics exposure in secondary peritonitis who will receive emergency surgery for acute abdomen without compromising clinical outcomes?*

1. We will collect background information of our hospital based antibiotic prescriptions in secondary peritonitis patients after laparotomy.
2. We will also explore the antibiotics prescriptions in secondary peritonitis in Taiwan using National Health Insurance Database (NHID).
3. To investigate the safety and feasibility of PCT-based algorithms to guide antibiotics treatment in secondary peritonitis after surgery.
4. To explore the potential biomarkers of sepsis such as plasma gelsolin and shingolipids using proteomic approaches.

**Study plan**

This is a pilot study to explore the feasibility and safety to use PCT-guided algorithm to guide post-op AB therapy in secondary peritonitis. We will enroll 30 patients who are diagnosed as secondary peritonitis with sepsis and needed surgical intervention in one-year period (between April 2012 and March 2013). The same kind of patients will be recruited from last year (between January 2010 and December 2011) as historical controls.

**Subject inclusion/exclusion criteria**

Patients ≥ 20 years who will receive emergency abdominal operations with sepsis due to secondary peritonitis are eligible for enrollment. Patients who meet one or more of the following criteria will be excluded: (a) patients who are moribund (life expectance not expected to be > 72 hours because of imminent death) (b) patients with liver cirrhosis, Child’s class B and C liver disease (c) patients with profound septic shock under treatment of high dose inotropic agents (d) pre-existing infection underwent antibiotics treatment (e) multiple traumatized patients with unstable hemodynamic status (f) patients or their family will not participate in this study (g) immunocompromised patients such as HIV infection, long term steroids treatment, post-chemotherapy (h) pregnant women.

**Definition**

Sepsis was defined according to the ACCP/SSCM consensus conference of 1992 [[15](#_ENREF_15)].

**Treatment plan**

All patients who fulfill the inclusion/exclusion criteria will receive empiric antibiotics treatment pre-operation and immediately post-operation. All patients received the first dose of antibiotics at the emergency department on diagnosis. After surgery, all participants received empirical antibiotic treatment (ceftriaxone 1 gram intravenous every 12 hours and metronidazole 500 mg intravenous infusion every 8 hours). Infection specialists blinded to this project will routinely review the justification of antibiotics treatment including choices and duration in every patient. The in-charge physicians will decide whether to discontinue antibiotics according to the algorithm and clinical conditions or extend the antibiotics treatment if clinically needed.

**PCT algorithm**

Antibiotic treatment was discontinued if clinical signs of infection improved and the PCT value was either <1.0 ng/ml or decreased to less than 80% of the initial pre-operative concentration over 3 postoperative days.

**PCT and biomarkers measurement**

PCT levels will be obtained pre-operation, post-operative day1, 3, 5, 7 by central lab in Chang-Gung Memorial Hospital (CGMH)-Keelung Branch. The additional levels will be ordered by physicians after one week if clinically needed. Besides, plasma samples drawn by patients on pre-operation, post-operative day1, 3, 5, 7, and before discharge will be stored at -80℃ freezer at 8^th^ floor in the operating room in CGMH-Keelung Branch until analysis of liquid chromatography coupled tandem mass spectrometry (LC-MS/MS).

**PCT-assays**

**Measuring Range:** The BRAHMS PCT KRYPTOR assay measures concentrations between 0.02 and 5000 ng/ml (0.02 to 50 ng/ml directly and up to 5000 ng/ml after sample dilution).

**Precision:** BRAHMS PCT KRYPTOR shows a high precision in the determination of serum/plasma PCT concentrations. The intra-assay CV and the inter-assay CV are 2-3% on the whole PCT concentration range.

**Analytical Sensitivity:** The detection limit calculated using the imprecision profile has been assessed as being 0.019 ng/ml with a probability of 95 %.

**Functional Assay Sensitivity:** The functional assay sensitivity (defined as the lowest analyte concentration that can be determined with an inter-assay CV < 20) has been assessed as being 0.06 ng/ml with a probability of 95%.

**Specificity**: The antibodies used in this assay show no cross-reaction with human calcitonin (up to 2.5 ng/ml), human katacalcin (up to 10 ng/ml), human α-CGRP and ß-CGRP (up to 4 µg/ml).

**Data collection**

In all patients, age, gender, diagnosis, co-morbidities, length of hospital and intensive care unit (ICU) stay, outcomes, and duration of antibiotic treatment will be recorded. Clinical bio-chemical data will be recorded, too. In addition, all microorganisms isolated and antibiotic substances applied will be documented.

**Measurements and observations**

In all patients, PCT was measured using a rapid sensitive assay with a functional assay sensitivity of 0.06 μg/L (Kryptor PCT; Brahms, Hennigsdorf, Germany) and an assay time of less than 20 minutes.

**Screening, baseline, and post-treatment follow-up**

Patients who are diagnosed as secondary peritonitis and needed surgical intervention will be assessed eligibility. Baseline markers will be obtained during the induction of general anesthesia. Post-treatment follow-up will last for one month after discharge.

**Serious adverse events**

Death from any causes is considered as a serious adverse event (SAE).

**Adverse events attributions**

ICU re-admission for any reason, disease-specific complications (i.e., persistence or development of intra-abdominal infections or abscess, and re-operation) and any intra-abdominal infectious complications in need of further antibiotics treatment after termination of antibiotics according to the studied PCT algorithms are defined as the adverse events (AE).

**Adverse event reporting**

The PI is responsible to report the occurrence of AE and SAE to the IRB promptly.

**Statistical methods**

Data analysis was performed using the R 2.12.1 (R Foundation for Statistical Computing, Vienna, Austria) software. In statistical testing, two-sided *p* value ≤ 0.05 was considered statistically significant. To answer the research question that “Does PCT-based algorithms to guide antibiotic therapy reduce the risk of antibiotics exposure in secondary peritonitis who will receive emergent surgery for acute abdomen without compromising clinical outcomes?”, the time to stop using antibiotics (in days) will be recorded as the primary end point. The same kind of patients will be recruited from last year (from June 2010 to May 2011) as historical controls.

The distributional properties of continuous variables were expressed as mean ± standard deviation (SD), interquartile range (IQR), and range, whereas categorical variables were presented as frequency and percentage. The Kaplan-Meier estimate of the survival curve for time to stop using antibiotics (in days) will be drawn. In univariate analysis, the baseline characteristics, potential prognostic factors for treatment failure of antibiotics, occurrences of serious adverse events (SAE), and the time to stop using antibiotics (in days) were compared between the study group and history controls using chi-square test, Fisher’s exact test, two-sample *t* test, Wilcoxon rank-sum test, and log-rank test as appropriate. Next, multivariate analysis will be conducted using Cox’s proportional hazards model to estimate the effects of PCT and prognostic factors on the time to stop using antibiotics (in days). If the characteristics between the study group and history controls so different that selection bias may occur, then propensity score analysis (PSA) will be applied to reduce selection bias.

To ensure the quality of analysis results, the model-fitting techniques for (1) variable selection, (2) goodness-of-fit (GOF) assessment, and (3) regression diagnostics will be used in regression analysis. Specifically, the stepwise variable selection procedure (with iterations between the forward and backward steps) will be applied to obtain the candidate final regression model. All the univariate significant and non-significant relevant covariates and some of their interactions are put on the variable list to be selected and the significance levels for entry (SLE) and for stay (SLS) are set to 0.15 or larger. Then, with the aid of substantive knowledge, the best final regression model is identified manually by reducing the significance levels to 0.05 corresponding to the chosen α level. Since the statistical testing at each step of the stepwise variable selection procedure is conditioning on the other covariates in the regression model, the multiple testing problem is not of concern. Any discrepancy between the results of univariate analysis and multivariate analysis is likely due to the confounding effects of the uncontrolled covariates in the univariate analysis. Both the GOF measure, adjusted generalized *R*^2^, and the GOF tests, Grønnesby-Borgan test, are examined to assess the GOF of the fitted Cox’s proportional hazards model. The generalized additive models (GAM) are applied to detect nonlinear effects of continuous covariates. And, the statistical tools for regression diagnostics such as verification of proportional hazards assumption, residual analysis, detection of influential cases, and check for multicollinearity are used to discover model or data problems.

**Subject withdrawal and discontinuation**

Patients and their families have rights to terminate this study at any time without any reason.

**Protocol deviations**

Protocol deviations are defined as prolonged antibiotics treatment when PCT algorithms suggest discontinuation of antibiotics if not clinically needed.

**Study registry**

This study will be registered on-line.

**Record retention**

The PI and co-PIs will responsible for record retention.

**References**

1. Solomkin, J.S., et al., *Diagnosis and management of complicated intra-abdominal infection in adults and children: guidelines by the Surgical Infection Society and the Infectious Diseases Society of America.* Clin Infect Dis, 2010. 50(2): p. 133-64.

2. *Guidelines for antimicrobial therapy of intra-abdominal infections in adults.* J Microbiol Immunol Infect, 2008. 41(3): p. 279-81.

3. Swenson, B.R., et al., *Choosing antibiotics for intra-abdominal infections: what do we mean by "high risk"?* Surg Infect (Larchmt), 2009. 10(1): p. 29-39.

4. Shine, B., F.C. de Beer, and M.B. Pepys, *Solid phase radioimmunoassays for human C-reactive protein.* Clin Chim Acta, 1981. 117(1): p. 13-23.

5. Raitakari, M., et al., *Distribution and determinants of serum high-sensitive C-reactive protein in a population of young adults: The Cardiovascular Risk in Young Finns Study.* J Intern Med, 2005. 258(5): p. 428-34.

6. Marshall, J.C. and K. Reinhart, *Biomarkers of sepsis.* Crit Care Med, 2009. 37(7): p. 2290-8.

7. Muller, B., et al., *Ubiquitous expression of the calcitonin-i gene in multiple tissues in response to sepsis.* J Clin Endocrinol Metab, 2001. 86(1): p. 396-404.

8. Harbarth, S., et al., *Diagnostic value of procalcitonin, interleukin-6, and interleukin-8 in critically ill patients admitted with suspected sepsis.* Am J Respir Crit Care Med, 2001. 164(3): p. 396-402.

9. Muller, B., et al., *Diagnostic and prognostic accuracy of clinical and laboratory parameters in community-acquired pneumonia.* BMC Infect Dis, 2007. 7: p. 10.

10. Bouadma, L., et al., *Use of procalcitonin to reduce patients' exposure to antibiotics in intensive care units (PRORATA trial): a multicentre randomised controlled trial.* Lancet, 2010. 375(9713): p. 463-74.

11. Schuetz, P., et al., *Effect of procalcitonin-based guidelines vs standard guidelines on antibiotic use in lower respiratory tract infections: the ProHOSP randomized controlled trial.* JAMA, 2009. 302(10): p. 1059-66.

12. Lee, P.S., et al., *Plasma gelsolin depletion and circulating actin in sepsis: a pilot study.* PLoS One, 2008. 3(11): p. e3712.

13. Puneet, P., et al., *SphK1 regulates proinflammatory responses associated with endotoxin and polymicrobial sepsis.* Science, 2010. 328(5983): p. 1290-4.

14. Wymann, M.P. and R. Schneiter, *Lipid signalling in disease.* Nat Rev Mol Cell Biol, 2008. 9(2): p. 162-76.

15. Bone, R.C., et al., *Definitions for sepsis and organ failure and guidelines for the use of innovative therapies in sepsis. The ACCP/SCCM Consensus Conference Committee. American College of Chest Physicians/Society of Critical Care Medicine.* Chest, 1992. 101(6): p. 1644-55.
